# Treatment of Sciatica

**Published:** 1871-07

**Authors:** J. Ernest Meire

**Affiliations:** N. Y.


### TREATMENT OF SCIATICA.

BY J. ERNEST MEIRE, M. D., N. Y.

The following treatment which I have adopted in three cases of sciatica of several years' standing, with the most gratifying results, I publish to the profession, believing by its strict observance we have the means of controlling and of eradicating this disease.

I shall give the history and its treatment of one case, it being the most severe.

Thomas Browning, aged 45, a farmer of western Maryland, presented himself at my office for treatment, having for the eighteen months previous suffered from sciatica. The entire right limb was *atrophied*, and he said he could *span it at the largest part of the thigh with one hand.* He was entirely incapacitated from attending to the duties of his farm, and could barely "hobble" about with the aid of a stick. He was very much discouraged, having previously to my seeing him been under the treatment of several physicians "without much benefit." On my giving him assurance that I could benefit him, he placed himself under my care on the 4th of April, 1870, and continued under treatment, at intervals, until July, when I saw him professionally for the last time.

The treatment was as follows:

He was cupped along the course of the sciatic nerve to the ancle, taking about eight ounces of blood. He was then ordered:

℞—Ol. tiglii gtt. iii.

Fiat in pil. No. 3.

Sig., one each night.

Should the pills operate too frequently their action can be easily checked with tinct. opii.

The following mixture was then given him, which I am confident has a very great controlling effect on the disease.

| | | |
|---|---|---|
| *℞—Quin. sulph. . . . | ℈ iss. | |
| Potassæ iodidi, . . . | ℥ ss. | |
| Vini colch, sem. fl. . . . | ℥ i. | |
| Aquæ dest., . . . | ℥ vi. | M. |

Sig., teaspoonful three times daily.

The cupping was repeated on the 8th and 23d of the same month, taking about the same quantity of blood as at the first operation. The mixture was continued at intervals up to July, but I should advise that there be no interruption in its administration until the termination of the treatment.

The result of the treatment in Mr. Browning's case I will give

*To prevent flocculent solution, the quinine and iodide of potassa should be dissolved in different vessels, or else a precipitate of the insoluble iodide of quinine will be thrown down and the medicine be of no effect.

in his own words: "Doctor, I am now able to attend to the duties of my farm, having myself plowed a large field, and my leg has regained its former size and strength, and I am entirely free from pain."

Before concluding this report I would recommend that the limb be subjected to a cold water douche for five or ten minutes, morning and night, both in winter and summer, for several months after complete restoration, and strict attention be paid to the bowels not permitting a day to pass without an operation.—[Med. Rec

